# A Pressure Sensing System for Heart Rate Monitoring with Polymer-Based Pressure Sensors and an Anti-Interference Post Processing Circuit

**DOI:** 10.3390/s150203224

**Published:** 2015-02-02

**Authors:** Yi Shu, Cheng Li, Zhe Wang, Wentian Mi, Yuxing Li, Tian-Ling Ren

**Affiliations:** Institute of Microelectronics and Tsinghua National Laboratory for Information Science and Technology (TNList), Tsinghua University, Beijing 100084, China; E-Mails: shuy11@mails.tsinghua.edu.cn (Y.S.); lic71317@gmail.com (C.L.); wangzhe.thu2012@gmail.com (Z.W.); miwentian@gmail.com (W.M.) qhlyx_1992@163.com (Y.L.)

**Keywords:** heart rate monitoring, flexible sensors, pressure sensors, anti-interference circuit

## Abstract

Heart rate measurement is a basic and important issue for either medical diagnosis or daily health monitoring. In this work great efforts have been focused on realizing a portable, comfortable and low cost solution for long-term domestic heart rate monitoring. A tiny but efficient measurement system composed of a polymer-based flexible pressure sensor and an analog anti-interference readout circuit is proposed; manufactured and tested. The proposed polymer-based pressure sensor has a linear response and high sensitivity of 13.4 kPa^−1^. With the circuit’s outstanding capability in removing interference caused by body movement and the highly sensitive flexible sensor device, comfortable long-term heart rate monitoring becomes more realistic. Comparative tests prove that the proposed system has equivalent capability (accuracy: <3%) in heart rate measurement to the commercial product.

## 1. Introduction

Heart rate has been an important factor in indicating patients’ health for quite a long time. As people are paying more and more attention on their health, long-term heart rate monitoring is of more importance to everyone as it’s a valuable indicator for early diagnosis of dozens of diseases. Furthermore, long-term heart rate monitoring is also essential for sports enthusiasts and professional athletes. According to these requirements, it is necessary to design and fabricate a device which is suitable for long-term heart rate monitoring and free from external disturbance. The wearing comfort, the reliability and the cost become three key issues for long-term heart rate monitoring for daily use.

Several conventional methods have already been developed to measure heart rate, most of which are based on electrocardiograms (ECGs) [1], photoplethysmography (PPG) [2], and the piezoelectric effect [3–5]. Other principles in heart rate measurement have also been verified by researchers [6–8]. Among these methods for heart rate monitoring, ECG is the most widely used. It is the standard diagnosis technique for heart disease in hospitals but the specialized equipment needed is clearly not suitable for household applications. Household heart rate monitors based on ECG technique have also been commercialized, but chest strap transmitters are usually needed, which is not appropriate for long-term use. Strapless wrist band ECG heart rate monitors have been developed these days. However, the contradiction between reliable electric connection and the comfort of wearing still has not been settled in a satisfactory way. Techniques for PPG require hard photoelectric modules which are tightly attached to the tissue with a certain penetration depth, for instance, the fingers. For the reasons above, these two kinds of heard rate monitor system are not appropriate for long-term ordinary daily use because of the inconvenience in user experience, especially at night. Monitors based on piezoelectric pressure sensor also have the similar limits.

## 2. System Design

In this work, a comfort, reliable heart rate monitor for long-term household use is proposed. Unlike the previous ECG, PPG and piezoelectric devices, a flexible pressure sensor with the capability of measuring heart rate by sensing the pulsation of the artery is used in the system. This solution is adopted based on the following considerations.

Since the sensor of the monitoring system is the only component which needs to inevitably be in contact with the human skin, trying to have a flexible sensor is crucial in realizing a comfortable, simple heart rate monitor. In this work, a robust, low-cost novel flexible pressure sensor has been proposed and fabricated. The polymer-based flexible sensor has no hard components which it can ensure its comfort and fitness for wearing all day in any situation.

Furthermore, to distinguish the tiny pressure changes caused by the artery pulse, the flexible pressure sensor’s sensitivity needs to be improved. Based on previous works in this area, a highly sensitive structure is proposed. The conventional structure with one piezoresistive layer is replaced by a novel structure with two piezoresistive layers. The contact interface of the two layers is modified with micro structures. These microstructures are realized with modern soft lithography technology and are treated as the key features for the sensor’s high sensitivity. In addition to that, for the specific morphology of the surface microstructures utilized, the pressure sensor device also has a perfect linearity performance.

However, due to the high sensitivity of the pressure sensor, noises may be induced by the wearer’s muscle movements. This inevitable daily movement can cause pressure changes between the wearer’s skin and the device. Such noises can easily make the monitor fail when heart rate counting. In order to solve this problem while maintaining a relatively low cost in signal processing, an Analog Signal Processing (ASP) system is proposed to process the signal and extract the heart rate information from it. The whole system is composed of a sensor subsystem and the ASP subsystem. The sensor subsystem is an elastic belt with the flexible pressure sensor attached on it. The tightness of the belt can be tuned in order to provide a proper pressure for measurement. The system also includes an ASP subsystem to process the pressure signals, a counter, an indicator, and a timing circuit which is used to stop the measurement.

### 2.1. Flexible Pressure Sensor

**Figure 1.**
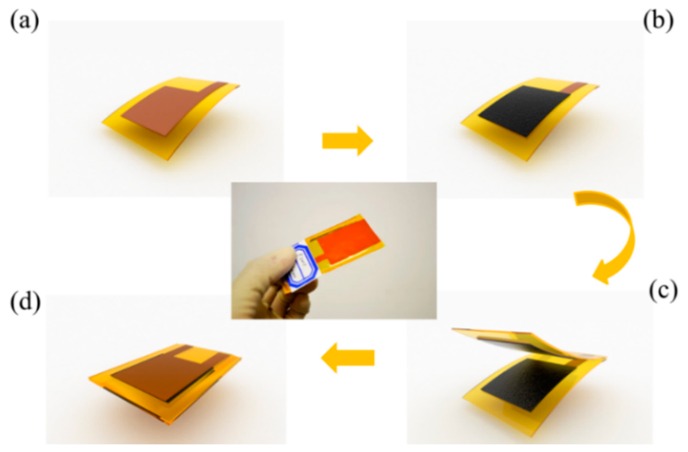
Assembly and packaging flow of the sensitive flexible pressure sensor. (**a**) A layer of patterned FCCL film is prepared as the electrode and package film; (**b**) The surface-modified piezoresistive film is adhered to the FCCL electrode; (**c**) Two assembled FCCL/piezoresistive composite films are placed face-to-face; (**d**) All layers are been packaged together by another layer of polymide (PI) bond-ply.

To realize the characteristic of flexibility in addition to the pressure sensing capability, a flexible piezoresistive carbon black/silicone rubber nanocomposite is adopted as the functional material [9–12]. For its stretchable, flexible properties and piezoresistive effect, this nanocomposite material has already been used in tactile sensors for robots to provide contact or grasping force feedback [13,14]. They are also some other sensors for mechanics that adopt the material for their piezoresistive properties and energy absorbing capabilities in preventing mechanical collisions [15,16]. In addition to that, all other materials used in device are flexible. Figure 1 shows a simple schematic diagram demonstrating the assembly and packaging steps used in fabricating the device.

Two layers of surface-modified piezoresistive polymer nanocomposite films are placed face-to face inside the device as the pressure sensing layer. These polymer films are packaged by another two layer of patterned Flexible Copper Clad Laminate (FCCL) films which act either as the package material or as the electrode of the device. To isolate this device from external moisture and contamination, another layer of PI bond-ply is used as the adhesion layer to ensure these four layers of material a good firm bond. These bond-ply layers have also been previously patterned by a laser cutting machine to save the space for the pressure sensing layer. The scale of the device’s sensing part is 15 × 30 mm^2^.

**Figure 2.**
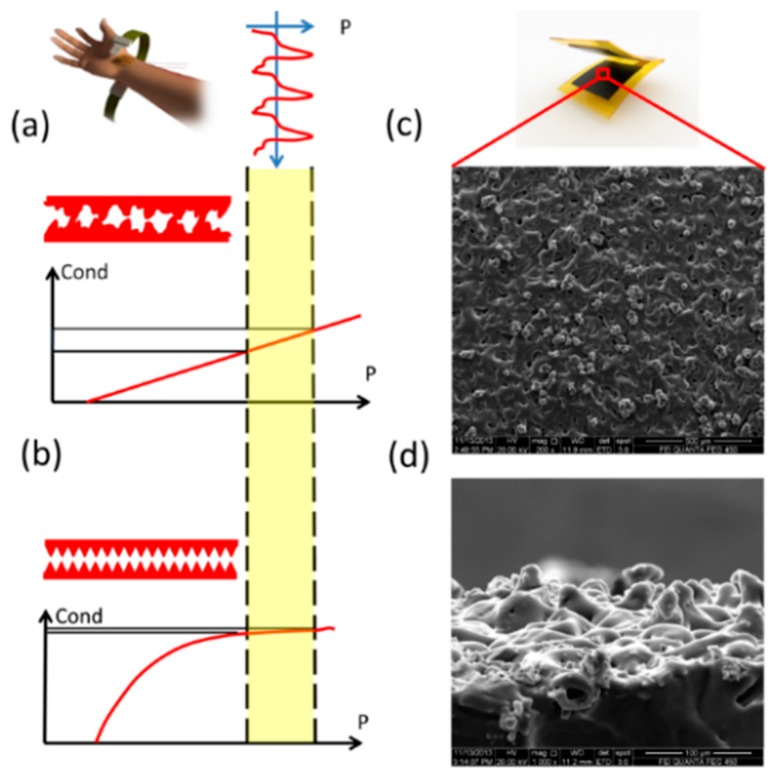
(**a**,**b**) demonstrate the comparison between the device performance with different contact surface profile; The SEM photos to the microbumps on the surface of carbon black/silicone rubber nanocomposite film in top view (**c**) and side view (**d**).

Since the human artery pulse in the wrist is weak and the excited pressure variation is not easy to sense, the flexible device’ sensitivity needs to be further improved. Several previous efforts have been made to promote the devices’ sensitivity in this research area [17–23]. A pressure sensor with record sensitivity with highly sensitive material with hollow polymer sphere in it has been proposed by Bao’s group [17]. Another type of research mainly utilizea tiny features’ very sensitive contact state to realize high sensitivity [18,19]. They usually have polymer-based micrometer scale pyramid structures to make the device electrically sensitive to external pressure loading. Devices of this type usually have a very sensitive electric response at the beginning when an array of unique pyramids gets contact to the opponent electrode. However, as the pressure goes higher, device’s response become dull as the pyramidal surface get saturated, as shown in Figure 2b.

A certain degree of pre-pressure needs to be maintained between the sensor and the human skin in order to ensure effective contact state and signal acquisition and the wrist artery pulse induced pressure varies (range < 3 kPa) based on this pre-pressure bias. Furthermore, the pre-pressure also changes with the differences between users when they fasten on the wrist belt. Consequently, the real effective span for the heart rate measurements will be around 8–18 kPa. Several previous works on flexible pressure sensors have reported sensitivity high enough for heart rate measurement. However, these high sensitivities only exist within a limited range around 0 Pa. As shown in Figure 2b, the slope of the device response decreases as pressure goes higher, like the sensitivity. When the pressure reaches the effective range, devices becomes so dull that it can’t respond to the artery pulse. As to the proposed novel device with full-scale high sensitivity (Figure 2a), artery pulse-induced pressure variation will make it respond intensively compared with previous ones.

To have this full-scale sensitive pressure response, a new flexible pressure sensing device have been proposed in this work. The surface of the nanocomposite film has been modified with micrometer scale bump structures randomly distributed on surface with certain variation in height and lateral size. Figure 2c–d shows the SEM photos of the microbumps from the top view and side view, respectively. Unlike the typical pyramid structures of the same size, the distributed tiny structures’ size makes them gradually touch with the opponent conductive surface along the whole pressure loading process so as to have a full scale high sensitivity. By surface profile measurement, the overall surface obeys the Gaussian Random distribution. By numerical simulation with MATLAB, the device response is expected to be linear.

Furthermore, the micrometer scale piezoresistive bumps on the surface are very sensitive to external pressure loading as the pressures are concentrated on these tiny structures. Large deformations of these piezoresistive structures lead to dramatic conductance variations of the device. Besides that, for the distributed bump size, the conducting paths are established gradually. The capability in conductance variation is even higher. Based on above statement, the device sensitivity is expected to be higher.

**Figure 3.**
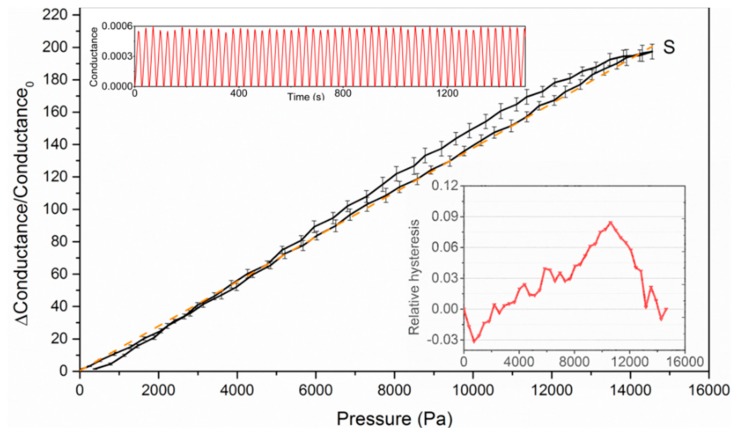
Device conductance variation performance test of the high sensitive pressure sensor along with the hysteresis test and repeatability test.

**Figure 4.**
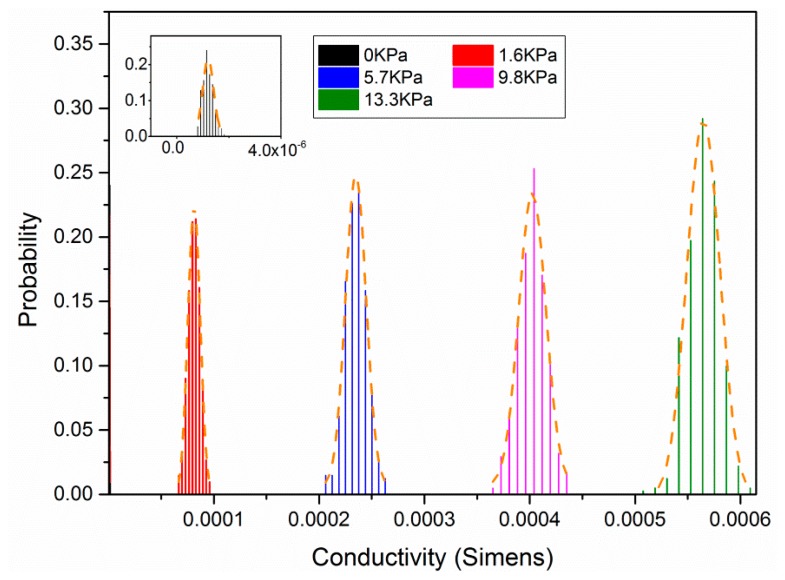
The statistical distribution of the measured conductance result at five different pressure levels.

The devices’ sensing capabilities were tested using a device test platform composed of a micropositioner, force gauge and the electric measurement part. All three of these parts are connected to a computer and controlled by a Labview software routine. Then, the device is tested for 100 cycles and the test results are plotted in Figure 3. The device responds linearly to the external pressure with the sensitivity around 13.4 kPa^−1^. A certain degree of hysteresis has been observed and the relative hysteresis does not exceed 9% over the full measurement scale. The device also shows an extraordinary stability and repeatability. Figure 4 shows the statistical distribution of the measured conductance result at five different pressure levels.

The temperature dependence of the fabricated device has also been tested, as shown in Supplementary file. However, as the readout circuit mechanism mentioned below, the temperature variation will not have an obvious influence on the system’s performance.

### 2.2. Materials and Methods

The piezoresistive polymer is prepared with the 107 silicone rubber (hydroxyl end-blocked dimethylsiloxane) and the super conductive carbon black (HG-3F: average diameter-12 nm) with the weight ratio of 100:8. An extra amount of *n*-hexane solvent is added into the mixture to promote the dispersion of carbon black particles in the silicone rubber. A subsequent process of 10 h ultrasonic treatment along with the fast stirring is carry out to ensure the carbon black’s sufficient dispersion. Before using, a proper amount of curing agent is added into the mixture (carbon grease) with a certain degree of stirring and ultrasonic treatment. Then, the carbon grease is spun coated on the GRD surface mould with the spin speed of 1000 rpm. After about 48 h of curing process under room temperature, the piezeresistive film on the mould is peeled off by hand and tailored to the proper shape and size to match the device design.

### 2.3. The Signal Processing Circuit

To make full use of the piezoresistive pressure sensor, an operational amplifier (op-amp) other than a Wheastone bridge [24] is used to transform the variation of the resistance into a voltage signal. This system is designed with the following principles. Firstly, it uses basic low-cost analog electronic components to lower the over-all cost. Secondly, it is designed to reduce noises and interferences caused by different sources. High-frequency noise is induced by the electromagnetic interference of the environment. The movement of human body, either intended or not, can also cause significant pressure changes in the sensor. This will result in undesired dc-drift of the signal which can be obviously observed before ASP circuits. Finally, after reducing the noises, the system converts the signal into a level signal, which is transferred into a low-cost digital counter.

**Figure 5.**
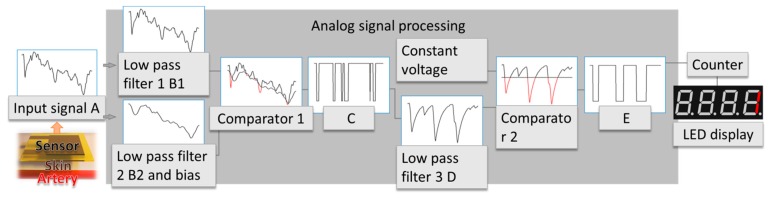
Block diagram of the heart rate sensor. The pulsation of the artery is transmitted to the sensor through skin, and generate voltage signal A. B1, B2, C, D, E are the signals inside the ASP module. B1 shows the signal generated from low pass filter 1, B2 shows the output of low pass filter 2. Comparator 1 generates C, which is the compare result of B1 and B2. The red part shows where B2 is higher than B1. D is generated by low pass filter 3. Comparator 2 generates E, which is the compare result of D and a constant voltage. Red parts show where the constant voltage is higher than D. E is used by the counter.

The flow chart of the ASP system is shown in Figure 5. The first op-amp transforms the pressure on the sensor into signal A. Each beat of the heart causes a short time of pressure change on the sensor, which leads to the change of resistance. This results in the variation of signal A. Signal A mainly contains heart rate information and high frequency noise and dc-drift. High frequency noise is introduced by the electromagnetic interference, and dc-drift is caused by human motion. Here the signal processing system is used to remove these two types of interference and extract heart rate information. An analog signal processing system is introduced, first two low pass filter with different cutoff frequency is used to process signal A. Low pass filter 1 generates signal B1. This filter is designed to filter noise with frequency much higher than the heart rate frequency (about 1–2 Hz) in order to reduce noise while preserving the heart rate information. This filter assures that B1 maintains the dc-drift and heart rate information. Low pass filter 2 generates signal B2 by removing the high frequency signal and add a voltage bias to signal A in order to compare with signal B2. It has a low cutoff frequency to only preserve the dc-drift of the original signal. Comparator 1 compare signal B1 with B2 and generate signal C. These series of processes make sure that signal C contains most of the heart rate information while staying nearly undisturbed by dc-drift. However, signal C still has too many glitches and cannot be used to count heart rate directly. Low pass filter 3 is used to reduce the glitches of signal C and generates signal D. Comparator 2 is introduced in order to compare signal D with a constant voltage. Signal E is generated as a result of the comparison of signal D and the constant voltage. Finally E only contains heart rate information and is used to count heart rate.

## 3. Integration and Test

**Figure 6.**
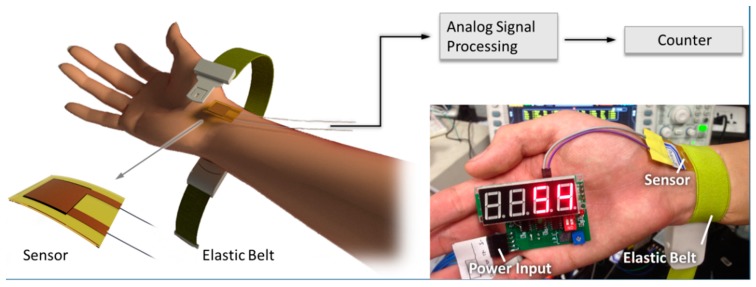
The test setup and the device. The test setup consists of a flexible pressure sensor, which is attached to the skin by an elastic belt. The ASP is used to process the signal and transmit it into the counter. Inset shows a view of the test setup, the module, power input, the elastic belt and the sensor.

The schematic of the device and the test setup in this experiment is shown in Figure 6. The whole system consists of an elastic belt which is used to attach the sensor onto the wearer’s wrist, the flexible pressure sensor and a module which contains the ASP system and the counter. This module is used to indicate the heart rate, and power input. The artery’s pulsation is transmitted to the human skin, normally this pressure change can be easily sensed if a fingertip is put onto a human’s wrist. Under this application situation, an elastic belt is used to fasten the pressure sensor on the wrist above the artery, so that the pressure sensor can be firmly attached to the wrist while sensing the pressure change. The elastic belt and the pressure sensor are both flexible, which ensures that the heart rate monitor is comfortable to wear. The change of the applied pressure will cause the resistance change of the pressure sensor. An amplifier is used to convert the change of the resistance into a voltage signal. Then the voltage signal is processed by the ASP subsystem in the module, as discussed previously. The ASP subsystem generates a level signal which contains heart rate information. Finally, this level signal is transmitted into the counter directly to measure the heart rate. The power input is used to power up the whole system.

## 4. Results

**Figure 7.**
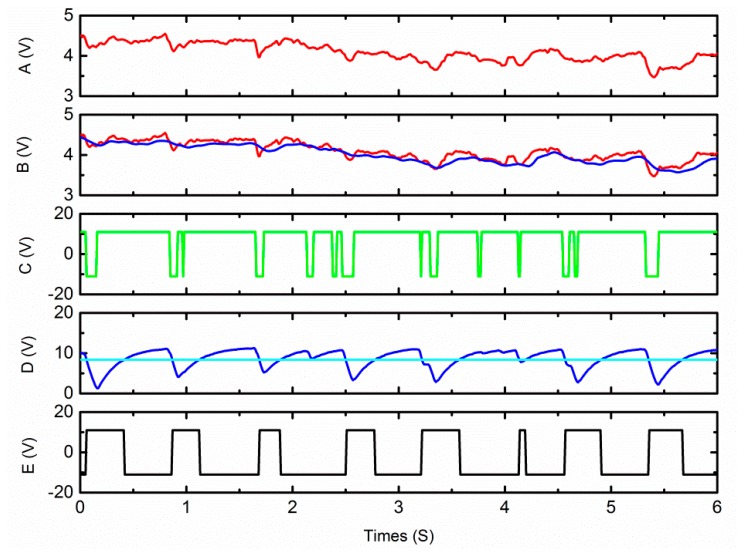
Input signal and output signal of the ASP subsystem. Traces’ name correspond to the output shown in Figure 1.

The heart rate monitor was tested on several researchers. An oscilloscope is used to capture the wave generated by the ASP subsystem while it is processing the signal. The waveform corresponding to the signal in Figure 5 is shown in Figure 7.

Signal A shows the voltage signal which is converted from the change of the resistance of the pressure sensor. There are eight small concave shapes on the waveform which indicate eight pulses, however this subtle heart rate signal cannot be used to count heart rate directly. B2 is the output of the low pass filter 2 with dc bias. This indicates that the dc-drift of the original signal is quite large, and even larger than pulse itself. However, testing results reveal that our ASP system can successfully remove the interference of the dc-drift. B1 is the result of low pass filter 1. Signal C is the compare result of B1 and B2. D is the output of the low pass filter 3. E is the result of the comparator 2, it is compressed proportionately and put into the counter. This result shows the monitor has good immunity to high level dc drift.

Several tests have been made to confirm the reliability of the monitor, see Table 1. Reference results are obtained from a commercial electronic sphygmomanometer (HEM-7052, OMRON, Kyoto, Japan). The average error is less than 3%.

## 5. Conclusions

A flexible, low-cost, small heart rate monitor comfortable enough for full-day wear is demonstrated. It is based on the pressure changes on the skin caused by the artery pulse. A sensitive flexible pressure sensor is used to sense the pressure change and an effective ASP system is used to extract heart rate information from the signal. This heart rate sensor has also been tested on several testees, and the results shows that this sensor is highly precise. The ASP system also shows its effectiveness in reducing the noise. Our work shows that based on this novel flexible pressure sensor and ASP system, a new way for heart rate monitoring can be realized. This work shows the possibility of full-day long-term heart rate monitoring.

## Supplementary Material



## Figures and Tables

**Table 1.** Result of the heart rate sensor and commercial electronic sphygmomanometer.

**Measurement Equipment**	**Heart Rate Sensor**	**Electronic Phygmomanometer**
Normal	66	64
	68	66
	70	70

After exercise	90	96
