# Supplementary material for: A Pressure Sensing System for Heart Rate Monitoring with Polymer-Based Pressure Sensors and an Anti-Interference Post Processing Circuit
Source: Sensors (Basel). 2015 Feb 2;15(2):3224–35. doi: 10.3390/s150203224 (PMC4367356; doi:10.3390/s150203224)
Supplement: Supplementary file 1 [file sensors-15-03224-s001.pdf]

OPEN ACCESS

sensors

ISSN 1424-8220
www.mdpi.com/journal/sensors

Supplementary Information

A Pressure Sensing System for Heart Rate Monitoring with Polymer-Based Pressure Sensors and an Anti-Interference Post Processing Circuit. *Sensors* 2015, *15*, 3224-3235

Yi Shu †, Cheng Li †, Zhe Wang, Wentian Mi, Yuxing Li and Tian-Ling Ren *

Institute of Microelectronics and Tsinghua National Laboratory for Information Science and Technology (TNList), Tsinghua University, Beijing 100084, China;
E-Mails: shuy11@mails.tsinghua.edu.cn (Y.S.); lic71317@gmail.com (C.L.);
wangzhe.thu2012@gmail.com (Z.W.); miwentian@gmail.com (W.M.)
qhlyx_1992@163.com (Y.L.)

† These authors contributed equally to this work.

* Author to whom correspondence should be addressed; E-Mail: rentl@tsinghua.edu.cn;
Tel.: +86-10-6279-8569.

The Temperature Dependence of the Flexible Pressure Sensor

A new temperature control device has been installed on our experimental setup and the temperature dependence test has been conducted with the results plotted in figure below.

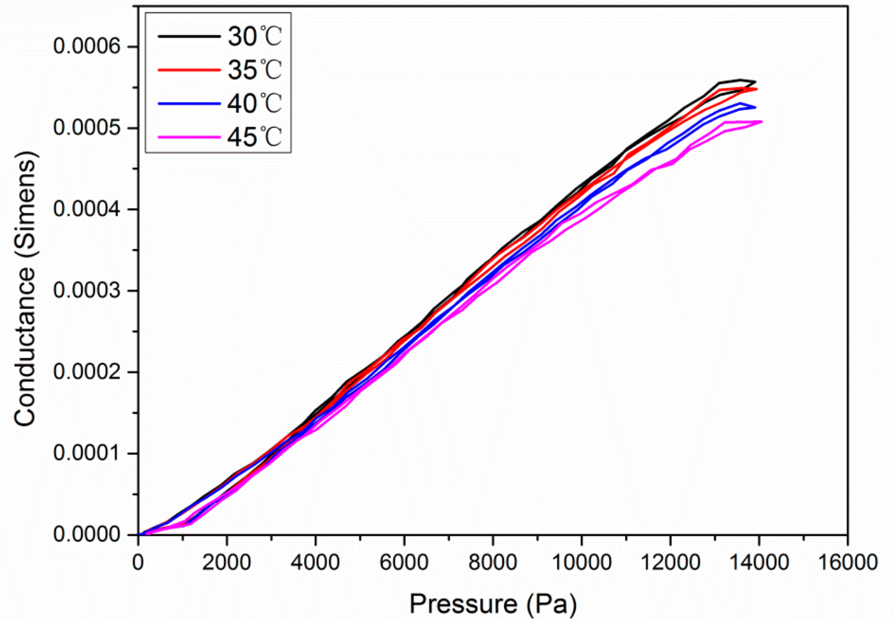

Figure S1. The temperature dependence of the flexible pressure sensor.

As the temperature rises from 30 °C to 45 °C, the device output conductance shows a small drift to lower values. The curve slopes at different temperatures and the fitted temperature coefficients are listed below.

Table S1. Performance variation under different temperature and the temperature coefficient.

30 °C	35 °C	40 °C	45 °C	Temperature Coefficient
0.4×10^{-4} Siemens/KPa	0.393×10^{-4} Siemens/KPa	0.378×10^{-4} Siemens/KPa	0.361×10^{-4} Siemens/KPa	-2.6×10^{-7} Siemens/(KPa·K)

© 2015 by the authors; licensee MDPI, Basel, Switzerland. This article is an open access article distributed under the terms and conditions of the Creative Commons Attribution license (http://creativecommons.org/licenses/by/4.0/).
